# Acta Societatis Medicæ Havniensis. Volumen II

**Published:** 1781-06

**Authors:** 


LONDON MEDICAL JOURNAL,

For JUNE 1781.

### SECTION I.

### BOOKS.

I. *Acta Societatis Medicæ Havniensis. Volumen* II. 8vo. Havniæ, 1779, 320 pages.

THE Medical Society at Copenhagen have publiſhed four volumes of their tranſactions; the two firſt under the title of *Collectanea*, and the two laſt under the preſent title. The volume before us contains the following papers:

1. *An account of the ſcarlatina that prevailed in* 1776 *and* 1777. *By* J. Eichel, M. D. *phyſician to the king for the province of Funen.*

The diſeaſe here deſcribed was a *ſcarlatina anginoſa*, the throat having been conſtantly more or leſs affected. Our author noticed three varieties of this fever. The firſt of theſe was attended with an univerfal eruption, and chiefly attacked very young fubjects. The firft fymptom of the difeafe was a painful fenfation in the fauces, which appeared inflamed and enlarged; to this, in the fpace ufually of twenty-four hours, fucceeded an eruption, which began commonly on the face and neck, and from thence fpread over the reft of the body. The pulfe was much quicker than would have been expected from the little heat and thirft of which the patients complained. The naufea, anxiety, &c. that preceded the eruption, went off foon after its appearance; but the quicknefs of pulfe increafed, efpecially at the beginning of the eruption. About the third day from the firft appearance of the eruption, the painful affection of the fauces began to diminifh, and the cuticle fcaled off in a dry fcurf. At this period of the difeafe, a moifture of the fkin was a favourable fymptom; on the contrary, if the fkin about that time was hot and dry, an alarming increafe of the inflammatory fymptoms ufually took place, and in fome patients terminated in an indolent fwelling of the parotid glands, or in a difcharge from the ears.

In the fecond fpecies the throat was more affected, and all the fymptoms more alarming.

The

The eruption was ſeldom to be perceived before the third day from the attack, and then only in ſome few parts of the body, chiefly about the joints, appearing in broad red ſpots of different ſizes. In the third ſpecies the throat only was affected, without any concomitant eruption.

II. *Of the malignant ſcarlatina that prevailed at Copenhagen during the ſummer of* 1777, *and the winter of* 1778. *By* Solomon Theophilus de Meza, M. D.

III. *An account of the angina and ſcarlatina that prevailed at Copenhagen in the years* 1777 *and* 1778. *By* Frederick Lewis Bang, M. D.

IV. *Practical obſervations on the epidemic ſcarlatina of* 1777 *and* 1778. *By* Urban Bruun Aaſkow, M. D.

The ſcarlatina anginoſa, after having been epidemic in Holſtein and Funen in 1776 and 1777, began to ſhew itſelf in Copenhagen about the month of July 1777; and the above three papers contain a hiſtory of this diſeaſe as it appeared in that capital, where it ſeems to have been attended with nearly the ſame variety of ſymptoms as that deſcribed by Dr. Eichel.

V. *A caſe of fiſtula in perinæo, by* Alexander Koelpin.—The patient whoſe caſe is here deſcribed, would not acknowledge that he had ever been infected with the venereal diseafe, but ascribed the origin of his complaint to a ftrangury that came on during a fever. When our author firft faw him in 1766, which was upwards of three years from the firft attack, the urethra feemed to be perfectly clofed, as neither bougies nor catgut could poffibly be introduced far into it, and all the urine the patient voided iffued from the fiftula in perinæo. From the great quantity of fediment the urine depofited, the bladder was fuppofed to contain a calculus; but no ftone could be felt by introducing the finger up the rectum. An incifion was made through the obftructed part of the urethra as far as the fiftula; but fo much was the canal contracted beyond this, that it was impoffible to introduce a catheter into the bladder. The day following, however, by introducing a piece of prepared fponge into the opening, the author dilated it fufficiently in the fpace of a few hours to admit a director, along which he carried his knife through the whole of the contracted portion of the urethra. He then removed the director, and in its ftead introduced a catheter, which after being properly fecured, was fuffered to remain for feveral days. At firft the urine was difcharged in part through the wound, and in in part through the catheter, but at the end of a week it paffed only through the former. The catheter was then removed, and after being cleaned, was again replaced as before. The introduction was now effected with eafe, and this operation of taking out, wiping and replacing the inftrument, was repeated at times for a fortnight, during which time, by the affiftance of compreffes of lint, only a very fmall proportion of urine flowed from the wound, the greater part of it paffing through the catheter.

As the patient now began to experience great inconvenience from the conftant ufe of the catheter, it was introduced only for about two hours every day, or occafionally when he wanted to make water. In this manner he proceeded to the end of the fecond month, when he began to void his urine without the catheter, though not without fome drops of it paffing through the wound. It was not till about four months after the operation, and after the patient had had an hernia humoralis and an abfcefs in the fcrotum, that the fiftula entirely healed, and the urine took its natural courfe.

VI. *A new method of treating uterine hæmorrhages after delivery. By* M. Saxtorph, M. D. [See our Journal for January, page 59.]

VII. *Of*

VII. *Of the morbus maculosus hæmorrhagicus. By* J. W. Gulbrand, M. D.—We have here an inſtance of a diſeaſe that has been very accurately deſcribed by Werlhof. The patient was a young man aged nineteen years, by trade a confectioner, of a fair complexion, florid cheeks, and ſlender make. Towards the end of the ſummer, and when the weather was rather cold, he was ſeized on a ſudden, and without any apparent cauſe, with a profuſe ſpitting of blood, attended with a ſlight cough, but without any concomitant fever or pain of the breaſt. The next day a ſurgeon took away a few ounces of blood, and recommended the uſe of nitre, an infuſion of arnica, cold barley water, &c. In ſpite of theſe remedies, however, the hæmorrhage continued. It was on the evening of the eleventh day from the attack that our author firſt ſaw him. He found him diſcharging a great quantity of a thin, dark coloured, fœtid blood at the mouth. His pulſe was ſoft, ſmall, and ſomewhat quick. Spots of a dark red colour, reſembling petechiæ, were perceptible in every part of the patient's body, but particularly on his legs and thighs, where they were larger and of a more livid hue. As the patient had been four days without going to ſtool, Dr. Gulbrand

Gulbrand began by prefcribing a clyfter and a folution of manna, after which he recommended a liberal ufe of a decoction of Peruvian bark, combined with vitriolic acid. The next day the patient had had three ftools, but his fymptoms were become more alarming. His pulfe was fmaller and quicker; his breathing fhorter; and the difcharge of blood by the mouth not abated. The night following he was more reftlefs than before; the petechiæ were more numerous, larger, and more livid; and his pulfe become hardly perceptible. Rhenifh wine was recommended along with the bark, but without effect, as he died on the 13th day.—Diffection threw no light on the caufe of the difeafe. All the abdominal vifcera were found, except the fpleen, which feemed to be larger than ufual, but without any morbid appearance. The pofterior furface of the right lobe of the lungs was flightly difeafed.

VIII. *A cafe of violent hyfteria cured by the Peruvian bark. By* John Henry Schoenheider, M. D.

In a cafe of hyfteria, attended with the moft formidable fymptoms, and where the paroxyfm returned every day at a ftated hour, our author fucceeded by means of the Peruvian bark given in in large doses, after the most powerful antispasmodics had been found ineffectual.

IX. *A treatise on putrid fever; which gained the prize offered by the College of Physicians at Copenhagen in the year* 1777. *By* F. L. Bang, M. D.

The views of the college were to determine, 1. The reason why putrid fevers are more frequent now than they were formerly; 2. The diagnostic symptoms that distinguish them from other acute fevers; and, 3. The best method of treating them.

To the first of these inquiries our author, after the most careful researches on the subject, ventures to reply that fevers of this class, instead of being more frequent, are probably less so now than they were formerly. In proof of this assertion he appeals to different writers, and to the reports of Frederick's Hospital at Copenhagen. His remarks on the diagnosis and treatment of putrid fevers are judicious, but contain nothing more than what is to be found in the best English writers on this subject.

X. *Of a singular stricture of the uterus, by which the head of the child was retained in an unnatural manner. By* M. Saxtorph, M. D.—The author relates the case of a woman, who was taken in labour labour in the ſeventh month of her pregnancy, and attended by a midwife. In extracting the fœtus the head was ſeparated from the body, and the former remained in the uterus. The midwife imputed this accident to a ſpaſmodic contraction of the neck of the uterus. After many fruitleſs attempts, during ſix hours, to finiſh the delivery, ſhe ſent for our author, who found the mouth of the uterus ſo dilated, that his hand paſſed eaſily into its cavity. The head of the child ſeemed to be incloſed in a kind of ſac, formed by a contraction of the uterus around its neck, ſo that no other part of it could be felt than the firſt vertebra colli projecting a little from the ſac into the cavity of the womb. The author's repeated endeavours to remove the head from this ſituation proving ineffectual, and the patient complaining much of pain, he deemed it prudent to withdraw his hand, and to preſcribe venæſection and twenty drops of tinctura thebaica. After a few hours, regular labour pains came on, and the head, being at length preſſed down into the vagina, was eaſily extracted.

The author cloſes his account of this caſe with ſeveral uſeful remarks. He obſerves, that the uterus oftentimes contracts ſo unequally,

 that that in attempting to turn a child he has more than once found the feet inclofed in a fac fimilar to the one juft now fpoken of, and that the placenta is frequently furrounded in a fimilar manner is, he adds, a well-known fact.

In cafes where the head of the fœtus is torn off in the uterus, and where no hæmorrhage or other alarming fymptom takes place, he very prudently advifes us to truft the delivery to nature.

XI. *A cafe of purulent cough cured by an abfcefs. By* J. W. Gulbrand, M. D.—The patient, a woman fifty-nine years old, after having been for fome time troubled with oppreffion at the breaft, cough, purulent expectoration, pain about the region of the liver, and an univerfal anafarca, was at length relieved by a large abfcefs a little below the right fcapula, which difcharged a great quantity of matter. Soon after this, iffues were opened in each of her legs; and by thefe means, together with the ufe of decoction of the bark and elixir of vitriol, fhe recovered in about five months. The author obferves, that a cafe fimilar to this is related by Van Swieten in the third volume of his commentaries. As an appendix to this paper, Dr. Gulbrand has added the following practical remarks, marks, viz. 1. He has feen an obftinate pain in the left knee, remaining after a rheumatic fever, carried off by an increafed fecretion of urine. 2. He finds a fcabious efflorefcence to be the moft certain crifis in the generality of fevers among the common people. 3. He has had occafion to treat a clonic fpafm of the ball of the right eye, which was occafioned by lightning, and refifted every remedy. 4. He has experienced excellent effects from clyfters of cold water in malignant fevers. 5. He has found a powder compofed of cream of tartar, kermes mineral, arum root and flowers of fulphur, ufeful as an alterative remedy. 6. In gouty cafes, and in glandular fwellings after fevers, attended with thick turbid urine, he has feen good effects from the ufe of the extracts of aconitum, cicuta, and belladonna. 7. After many trials he is far from being convinced of the efficacy of quaffia. 8. He had occafion to fee a young man, who foon after having been cured of the itch by the *ung. e fulphure*, was attacked with a difficulty of breathing fo as to be unable to lie down in his bed, and at the fame time had an œdematous fwelling of the left arm, with an irregular pulfe. The patient died; and on diffection, the whole of the left lobe of the lungs was converted as it were into a ſteatomatous tumour, that adhered to the pleura and ribs. 9. In another patient, a dropſical boy twelve years old, who had been cured of the itch in the ſame manner, our author found the right kidney changed into a ſimilar tumour. 10. He has ſeen intermittent fevers cured by *pulv. rad. calami aromatici*, after the Peruvian bark had been tried without effect.

XII. *Of the cure of the venereal diſeaſe in children. By* J. H. Schoenheider, M. D.

The author obſerves, that the diſeaſe commonly appears about the third or fourth week after birth. The firſt marks of it are uſually red or blue ſpots about the anus and pudenda, and ſometimes on the forehead. The mode of treatment he recommends, is to give the patient one grain of calomel with two grains of ſugar, and four of magneſia, daily. In very young children, twelve doſes are ſaid to be in general ſufficient; but he has ſometimes found it neceſſary to preſcribe forty. He has never ſeen a ſalivation excited by this courſe; a circumſtance he aſcribes to the frequent looſe ſtools to which children are ſubject. Sometimes, however, he has ſeen convulſions take place; but ſuch ſymptoms, he obſerves, were generally ſoon removed by by clysters, and by anointing the ſpine with the *unguentum nervinum* Pharm. Dan.

XIII. *A caſe of venereal conſumption cured by mercurial frictions.* *By* Sol. Theoph. de Meza, M. D.

We have already given our readers the whole of this paper in our Journal for February, page 129.

XIV. *Practical obſervations on the connexion of intermittent fevers with other diſeaſes.* *By* Fred. Lewis Bang, M. D.—The author of this paper preſents us with ſeveral remarks on the diſeaſes that accompany or are produced by intermittents, ſuch as topical pains, cough, diarrhœa, obſtructions of the liver, and dropſy. When the laſt of theſe takes place, he has generally found the moſt efficacious remedy to be cream of tartar, taken in doſes of two ſpoonfuls three times a day. In one caſe, however, in which a dropſy of the legs, thighs, and abdomen was brought on by an intermittent of eleven weeks ſtanding, the dropſy ſeemed to gain ground during the uſe of this remedy, ſo that our author judged it prudent to lay it aſide, and ſubſtitute the bark in its ſtead; by which means he had the pleaſure to ſee both the intermittent and dropſy ſpeedily removed. In another patient, a lady lady fifty years old, who had been troubled with an anafarca three years previous to the attack of the intermittent, both difeafes were cured in the fame manner.

XV. *Of an uncommon difeafe of the liver, cured by mercury. By* J. Clem. Tode, M. D.—We have here the cafe of a patient forty-five years old, who was feized in the Eaft Indies with fymptoms of an obftruction of the liver, which were foon removed, and he continued well till fome time after his return to Europe, when, without any apparent caufe, he was again attacked with the fame complaint. He breathed with difficulty, complained of a fenfation of weight and heat in the right hypochondrium, and had a hard painful fwelling at that part of the abdomen. To thefe fymptoms were added, flight fever, lofs of appetite, dejection of fpirits, and difturbed fleep. No yellow tinge was obfervable in the fkin, eyes, or urine; but the tumour in the hypochondrium enlarged, grew more painful, and to the touch feemed to afford marks of a fluctuation. In this ftate recourfe was had to mercury. Mercurial unction was rubbed upon the tumour, and calomel was given internally, combined with rhubarb and foap, according to the method mentioned by Dr.

Lind.

Lind. Within a few days the belly became more lax, and he voided much bile with his ſtools, an effect which no purgative remedies had before been able to produce. At the end of a fortnight all the ſymptoms were greatly diminiſhed; in the third week a gentle ſalivation was excited, ſo that the patient ſpit about half a pint daily. The doſes of mercury during the fourth and fifth weeks were gradually leſſened. A few gentle purgatives were preſcribed towards the end of the courſe, and the patient is now well.

XVI. *Practical remarks on hepatitis. By* U. B. Aaſkow, M. D.—In April and May 1778, ſeveral caſes occurred to the author, of hepatitis combined with a ſlight degree of jaundice. The diſeaſe was attended with fever which increaſed towards evening; colic pains, in ſome patients, accompanied with vomiting; a pain under the falſe ribs, on the right ſide, extending to the pit of the ſtomach, which was likewiſe painful when touched; cough, at firſt dry, but about the fourth day attended with a copious expectoration of yellowiſh mucus, now and then ſlightly ſtreaked with blood. The face, which was pale at the beginning, began about the third day to have a yellowiſh tinge. The urine, from being pale, pale, was obferved about the fourth day to depofit a bilious fediment. On the feventh day, which our author fuppofes to have been critical, the patient generally experienced relief, by having a flight difpofition to fweat, loofe ftools, and an increafed expectoration, which continued four or five days, when all the fymptoms ufually difappeared. The method of cure adopted by Dr. Aafkow was fimple, and in general fuccefsful. He began with venæfection, which was repeated if the pain did not foon abate. He was careful to keep the body conftantly lax; and for this purpofe prefcribed manna, honey, tincture of rhubarb, whey, &c. When the fever began to decline, he gave decoction of the bark, with rhubarb or oxymel of fquills. In two cafes where the pain continued obftinate, blifters applied near the feat of the pain had a good effect; and in one patient, whofe complaints were more troublefome and of much longer duration than the reft, recourfe was had to mercury, which excited a falivation that continued upwards of a month, and effected a cure. This laft cafe is related by the author at full length.

XVII. *A cafe of retroverted uterus. By* John Philip Rogert, M. D.—The fubject of this paper was a woman forty-four years old, who was was ſeized while at work with violent pains in the lower part of her abdomen, which ſhe aſ-cribed to colic. The day following ſhe com-plained of iſchuria; but being unwilling to apply for medical aſſiſtance, ſixteen days elapſed before our author ſaw her. On being told that her menſes had diſappeared about ten weeks before, he attributed her preſent complaints to pregnancy. On inquiry, a large, round, hard, and immoveable body was felt in the pelvis, but no veſtige of the os uteri could be diſtin-guiſhed. The belly was tenſe and painful. With ſome difficulty a catheter was introduced into the bladder, and two or three pints of urine were evacuated. The author was then able to diſcover the os uteri, concealed as it were under the pubis, and by introducing his hand into the vagina, and puſhing the fundus uteri upward and a little to one ſide, in order to avoid the prominence of the ſacrum, the uterus reſumed its natural poſition. The night following this operation, the patient had a ſlight diſcharge of blood from the uterus, which ſeemed to threaten abortion; but by keeping her quiet, and in bed for a few days, ſhe recovered and went her full time, at the end of which ſhe was happily de-livered.

XVIII. *An account of a peculiar tumour of the ovarium. By* M. Saxtorph, M. D.—A lady who had borne two children, was taken, when on the road and in a carriage, with a great deſire to make water. She retained it for ſome hours thro' modeſty; and then, when ſhe endeavoured to void it, was ſeized with violent pains and convulſions. At length ſhe evacuated her urine, and the convulſions ceaſed; but a few days after, feveriſh ſymptoms came on, attended with pain and a difficulty in making water. Theſe ſymptoms were occaſioned by a prolapſus uteri, which our author reduced by the preſſure of his hand. About a year afterwards as ſhe was riding in a coach the complaint returned, and was reduced in the ſame manner as before; but from that time ſhe conſtantly complained of a ſenſation of weight on the right ſide of the region of the pubis, and it was not long before a tumour was perceptible in that part. At length the right thigh ſwelled to an enormous bulk, became gangrenous, and the patient died. On diſſection, the right ovarium was found enlarged to the ſize of a child's head, and filled with a ſebaceous matter, in which was a conſiderable quantity of twiſted hair.

XIX. *Of the cure of an aſcites of five years ſtanding.*

*ſtanding*. *By* S. T. de Meza, M. D.—We have here an account of a woman forty years old, who, after thrice undergoing the operation of the paracenteſis, was at length cured by taking the *oxymel colchici* in the manner recommended by Baron Storck. The author informs us, that the fluid diſcharged at the third tapping had a purulent appearance, and that the patient afterwards became hectic, and voided little or no urine. He aſſures us, however, that in a few days after ſhe began to take the *colchicum*, the diſcharge by urine increaſed conſiderably, and that by perſevering for ſome time in the daily uſe of a few tea-ſpoonfuls of the remedy, all her ſymptoms gradually diſappeared.

XX. *An account of a tertian attended with remarkable ſymptoms*. *By* P. C. Abildgaard.—An hyſterical woman, forty years old, was attacked with a tertian, the ſecond paroxyſm of which was accompanied by a loſs of voice that continued ten hours. The third fit, however, produced a very different effect; for inſtead of aphonia, the patient was ſeized with what our author calls a *metro-mania*, or rage for reciting verſes, which ſhe did *extempore*, having never before had the leaſt taſte for poetry. When the fit was over ſhe became ſtupid, and remained ſo till the return of the paroxyſm, when ſhe reſumed her poetical powers again. After the ſixth paroxyſm, the bark, of which ſhe had taken half an ounce between each fit, not having proved efficacious, and the author, on account of her diſordered imagination and a troubleſome coſtiveneſs ſhe laboured under, fearing to increaſe the doſe of it, thought proper to lay it aſide, and in its ſtead preſcribed an emulſion of gum ammoniacum and aſafœtida, which in a few days removed the complaint.

XXI. *A caſe of exceſſive intoxication. By Henry Calliſen.*—A ſailor, thirty years old, after drinking immoderately of brandy, was left by his companions all night in the open air in autumn. The next morning he was brought to the Naval Hoſpital with all the ſymptoms of apoplexy; but by repeated bloodletting, and by getting emetic tartar into his ſtomach ſo as to excite vomiting, he recovered his ſenſes in about twenty hours.

XXII. *An inſtance of death occaſioned by the power of imagination. By* S. T. de Meza, M. D.—The diſeaſe which our author aſcribes to the power of imagination, ſeems to have been a phrenſy that originated from ſome other very different cauſe. The patient, a Jew, of a ſuper-

ſtitious,

ſtitious, melancholy diſpoſition, became ſuddenly furious. Emetics, venæſection, bliſters, and evacuations were tried without effect, and on the third day from the attack the patient died.

XXIII. *Three inſtances of unexpected recovery. By* J. H. Schoenheider, M. D.—The firſt of theſe is a caſe of midwifery in which the arm preſented. After replacing it, the author attempted to go up for the feet in order to turn the child; but not being able to do this, and the patient being exhauſted by the pains, he thought it prudent to deſiſt for a time, and recommended venæſection, reſt, and an antiphlogiſtic regimen. The next morning he was ſurprized to find that the woman had been delivered of a dead child without any aſſiſtance. The next is a caſe of ſtrangulated hernia, which after having been attended with the moſt alarming ſymptoms for three weeks, terminated favourably. The remedies employed were Peruvian bark, laxative clyſters, and cold applications to the hernia.—The third is a caſe of curvature of the ſpine, that came on during an acute diſeaſe in a youth of thirteen years, and was cured by his ſuſpending himſelf every day by his hands.

XXIV. *Medico-practical remarks on the uſe of leum aſphalti in phthiſis. By* F. L. Bang, M. D.

—Thirty

—— Thirty cafes of phthifis are related in which this remedy was adminiftered in dofes of eight or ten drops twice a day. In thirteen of them it proved fuccefsful; in the other twenty-two, this and every other medicine failed. From its effects in thefe cafes the author concludes, that it acts by promoting expectoration. He has generally found it moft beneficial in recent cafes, but he has feen it cure even when the difeafe has been of a year's ftanding. He affures us it proves equally efficacious, whether the difeafe originates from hæmoptyfis, a neglected cold, or peripneumony; but in phthifis attended with hoarfenefs, dry cough, or glandular fwellings, he has rarely feen it of ufe.

XXV. *Of the efficacy of an iffue in a violent ophthalmia. By* J. G. Gulbrand, M. D.—The cafe here defcribed occurred after the fmall-pox. Blifters, evacuants, faturnine and other topical applications were tried without effect, and the inflammation increafed fo much that matter was perceptible behind the cornea. In this alarming ftate an iffue was made in the patient's arm, and this, together with a cooling diet and occafional purges of jalap and calomel, effected a cure in about three weeks.

XXVI. *Remarks on the hypochondriacal difeafe, and*

*on*

*on the uſe of leeches in it. By* J. H. Schoenheider, M. D.—After ſome few prefatory obſervations on the conſent that prevails between the alimentary canal and the reſt of the ſyſtem, particularly in hypochondriacal patients, the author relates the caſe of a man, forty-five years old, who in his youth had been ſubject to profuſe hæmorrhage at the noſe, but was now become low-ſpirited, coſtive, and ſubject to flatus, vertigo, &c. He was now and then ſubject to hæmorrhoids, but the diſcharge was ſmall, and did not relieve him. Venæſection was tried, but without any better effect. At length ſix leeches were applied around the anus; they produced a copious flow of blood, which continued for ſeveral days, after which the coſtiveneſs and other ſymptoms were effectually removed.

XXVII. *Several remarkable caſes in ſurgery. By* Henry Calliſen.—The firſt of theſe is the caſe of a female patient, who laboured under all the ſymptoms of a ſtrangulated hernia femoralis. On the fourth day of the incarceration the menſes appeared, and the ſymptoms abated conſiderably; but returned again the day following, when the author thought it adviſeable to perform the operation uſual in ſuch caſes. On cutting through the ſkin, the tumour which he he had ſuppoſed to be the hernia, was found to be a glandular ſwelling ſeated in the cellular membrane. Beyond this, however, a ſmall hernia was diſcovered between the pectinalis and the tendon of the pſoas, which was eaſily reduced by dilating the ligamentum Fallopii.— The ſecond is likewiſe a caſe of hernia femoralis. On performing the operation, no appearance of a hernia could be diſcovered below the ligamentum Fallopii; but above it, the aponeuroſis was diſtended to the ſize of a pigeon's egg. An inciſion was made into it, and a piece of inteſtine, violently inflamed, found in the ſac. The cavity of this ſac communicated with the abdomen by a very ſmall opening, through which the inteſtine was eaſily reduced. The ſymptoms were relieved by this operation, but ſoon returned again with great violence, and produced death in twenty-four hours. On diſſection, another hernial ſac was diſcovered under the ligamentum latum, extending upwards towards the pſoas muſcle, and containing a portion of inteſtine in a gangrenous ſtate.—The third is a caſe of coſtiveneſs, which our author aſcribes to a palſy of the inteſtines, brought on by retrocedent gout. The diſeaſe proved fatal in nine days; and on diſſection, the *inteſtinum*

*cæcum*

*cæcum* was found enormoufly diftended, fo as to occupy almoft the whole cavity of the abdomen.—The fourth is a cafe of fatal hæmorrhage from the anus. The patient, who had been for three months labouring under a quartan, was feized, previous to a paroxyfm, with a violent pain in the left hypochondrium, accompanied with vomiting. The abdomen foon became diftended; and in about a quarter of an hour blood flowed from the anus, and the patient died convulfed, before the author, who was immediately fent for, could reach him. On diffection, the fpleen, which was very large and flaccid, was found adhering to and communicating by a fmall opening with the ftomach, which as well as the whole inteftinal canal, was filled with coagulated blood. The fifth and laft cafe affords an inftance of the vertebræ of the neck being luxated, without proving fatal. In diffecting a dead body, the author found a real anchylofis of the bodies, and articular proceffes of the third and fourth cervical vertebræ, both which were confiderably diftorted.

